# Race and geography impact validity of maximum allowable standing height equations for para-athletes

**DOI:** 10.1038/s41598-024-56597-y

**Published:** 2024-03-19

**Authors:** Brian S. Baum, Constance Man

**Affiliations:** 1 Cambridge, USA; 2 MIT Lincoln Laboratory, 244 Wood Street, Lexington, MA 02421 USA

**Keywords:** Bone quality and biomechanics, Physiology, Anatomy

## Abstract

World Athletics use maximum allowable standing height (MASH) equations for para-athletes with bilateral lower extremity amputations to estimate stature and limit prosthesis length since longer prostheses can provide running performance advantages. The equations were developed using a white Spanish population; however, validation for other races and geographical groups is limited. This study aimed to determine the validity of the MASH equations for Black and white Americans and whether bias errors between calculated and measured stature were similar between these populations. Sitting height, thigh length, upper arm length, forearm length, and arm span of 1899 male and 1127 female Black and white Americans from the Anthropometric Survey of US Army Personnel database were input into the 6 sex-specific MASH equations to enable comparisons of calculated and measured statures within and between Black and white groups. Two of 12 MASH equations validly calculated stature for Black Americans and 3 of 12 equations were valid for white Americans. Bias errors indicated greater underestimation or lesser overestimation of calculated statures in 10 equations for Black compared to white Americans and in 2 equations for white compared to Black Americans. This study illustrates that race and geography impact the validity of MASH equations.

## Introduction

World Athletics is the international governing body for athletic and para-athletic competitions, which establishes rules and regulations to provide fair and meaningful competitions that are as inclusive as possible^1^. Para-athletes with bilateral lower extremity amputations do not have a fully intact limb as a reference to determine their prosthetic length, so they have the unique ability to artificially increase their leg lengths and therefore their stature by wearing longer prostheses. This can provide unfair running performance advantages over runners with amputations who do not and potentially over runners without an amputation. Increased leg lengths can enable greater steady-speed, fatigue-resistant sprinting by increasing step lengths and ground contact lengths^2–6^. Longer legs may have speed disadvantages such as decreased step frequencies^7,8^, but overall, evidence supports that increasing prosthetic length allows para-athletes to achieve faster top running speeds^3,9,10^. Consequently, World Athletics implemented strict guidance on prosthesis use to provide fair competition for para-athletes and ensure the use of prostheses may go only as far as is necessary to preserve the fundamental nature, values, and meaning of the sport^1^.

The maximum allowable standing height (MASH) rules were established by the International Paralympic Committee to provide equitable competitions by limiting the length of the prostheses that athletes can use according to their predicted maximum height^11^. Six predictive equations for each biological sex, males and females, are currently used to determine the MASH for athletes with bilateral lower extremity amputations, regardless of race or nationality. These equations were developed based on proportionality between stature and body segment lengths, and a combination of sitting height, upper limb lengths, and lower limb lengths were used to generate stature estimations. Five of the 6 sex-specific MASH equations were developed by Canda and calculate stature with high correlation coefficients (R^2^ > 0.922 for males and R^2^ > 0.892 for females) and low root mean squared error (RMSE < 2.97 cm for males and RMSE < 2.34 cm for females)^12^. The remaining equation for each sex included only sitting height and was based on body proportion ratios generated by Contini^13^. The selection of the applicable equation for each athlete is based on the athlete’s available intact body segments, which may include the thigh, upper arm, and/or forearm.

The proportionality between stature and body segment lengths is influenced by numerous extrinsic and intrinsic variables. These extrinsic variables include physical factors such as distance from equator^14,15^, temperature^15–20^, humidity^16,21^, pathogen loads^22^, environmental resource availability^23^, and socioeconomic factors such as wealth^24,25^, education^26^, and occupation^27^. Intrinsic variables such as genetics^27–30^, biological sex^31,32^, age^31,32^, and race^33,34^ have likewise been observed to impact proportionality. The potential impact of geography and race on MASH equations is particularly important as World Para Athletics competitions include athletes from around the globe with 103 countries participating in the 2023 World Para Athletics^35^. Prior studies have suggested the need for race- and population-specific stature estimation methods because racial differences in limb proportions cause varying accuracy for multiple stature estimation equations^36,37^.

The currently used MASH equations were developed using a white Spanish population^12^ and were compared to various stature estimation equations in white Australian and Asian Japanese populations^38^. While only three countries are represented and relatively small populations were used to compare the equations in Australia (N = 30, 15 females) and Japan (N = 31, 15 females)^38^, the currently used MASH equations were determined to be the most accurate for estimating stature. However, to ensure the accuracy of the MASH equations for a specific race or geographical group, the equations must be validated specific to that population, thus additional studies are warranted.

Numerous studies support that Black and white people have different body segment proportions where Black people have relatively shorter trunks and longer limbs^32–34,36,37,39–44^. This emphasizes the importance of validating the MASH equations for a Black population. Additionally, the United States is consistently well represented in World Para Athletics competitions, but how the MASH equations may apply to Americans is unknown. Validating the MASH equations for Black and white American populations will improve our understanding of the generalizability of these equations to people from different races and geography and support global equity and fairness in para-athletic competitions.

The aim of this study is to determine if existing MASH equations validly calculate stature in Black and white populations from the United States. This study has 3 hypotheses: (1) the existing MASH equations will accurately calculate stature in Black Americans with no difference compared to their measured stature, (2) the existing MASH equations will accurately calculate stature in white Americans with no difference compared to their measured stature, and (3) the bias error, or difference, between calculated and measured stature for Black Americans will be similar to white Americans.

## Results

A total of 1959 males and 1145 females from the Anthropometric Survey of US Army Personnel (ANSUR II) database^45–47^ met the inclusion criteria. Sixty males and 18 females were identified as outliers and were removed from the study population resulting in 1899 males and 1127 females included for analysis.

Table 1 provides the study population’s age, mass, stature, and BMI values. No differences existed between Black and white males or females for these variables (*p* > 0.05). Body segment lengths and segment length to stature ratios are presented in Table 2. For males, thigh length, upper arm length, forearm length, and arm span lengths and length to stature ratios were greater in the Black group (*p* < 0.001 for all); however, sitting height and sitting height to stature ratio (*p* < 0*.*001) were shorter in Black compared to white males. For females, no difference existed between races for upper arm length (*p* = 0*.*079). Thigh length, forearm length, and arm span were longer in Black compared to white females (*p* < 0.001 for all), while sitting height was shorter for Black females (*p* < 0.001). Black females had greater body segment length to stature ratios for thigh length, upper arm length, forearm length, and arm span (*p* < 0*.*001 for all) and smaller sitting height to stature ratios (*p* < 0*.*001) compared to white females.

**Table 1:** Study population description.

	N	Age (years)	Mass (kg)	Stature (cm)	BMI (kg/m^2^)
Black male	302	25.5 ± 4.6	78.38 ± 9.52	175.6 ± 6.2	25.39 ± 2.57
White male	1597	25.1 ± 4.5	79.33 ± 10.05	176.3 ± 6.1	25.50 ± 2.74
Black female	412	25.2 ± 4.6	65.81 ± 9.43	163.1 ± 6.4	24.67 ± 2.82
White female	715	24.8 ± 4.4	65.88 ± 8.74	164.0 ± 5.6	24.47 ± 2.69

Values are mean ± 1 standard deviation.

*BMI* body mass index, *N* number of participants.

**Table 2:** Body segment lengths, in cm, and proportionality calculated as body segment length to stature ratios.

	Male		Female	
	Black	White	Black	White
Sitting height (cm)	89.3 ± 3.0*	92.6 ± 3.1	84.1 ± 3.2*	87.0 ± 2.8
Thigh length (cm)	44.9 ± 2.7*	43.2 ± 2.5	41.7 ± 2.3*	40.6 ± 2.1
Upper arm length (cm)	33.8 ± 1.5*	33.5 ± 1.6	31.4 ± 1.6	31.2 ± 1.5
Forearm length (cm)	28.1 ± 1.4*	26.6 ± 1.3	25.1 ± 1.5*	23.8 ± 1.2
Arm span (cm)	186.4 ± 7.7*	180.6 ± 7.6	170.0 ± 8.0*	165.1 ± 6.9
Sitting height ratio	0.509 ± 0.013*	0.525 ± 0.011	0.516 ± 0.012*	0.531 ± 0.011
Thigh length ratio	0.255 ± 0.012*	0.245 ± 0.011	0.256 ± 0.010*	0.248 ± 0.009
Upper arm length ratio	0.193 ± 0.006*	0.190 ± 0.006	0.192 ± 0.006*	0.190 ± 0.006
Forearm length ratio	0.160 ± 0.006*	0.151 ± 0.005	0.154 ± 0.006*	0.145 ± 0.005
Arm span ratio	1.061 ± 0.026*	1.024 ± 0.024	1.042 ± 0.027*	1.007 ± 0.022

Values are mean ± 1 standard deviation.

*Significant difference (*p* ≤ 0.001) between sex-specific black and white groups.

Stature was calculated using each of the sex-specific MASH equations and compared to measured stature (Table 3). For males, the main effect of stature showed a significant difference in mean stature and calculation method (*F*_(2.81, 5328.31)_ = 519.97, *p* < 0*.*001), and a significant interaction between race and stature existed (F_(2.81, 5328.31)_ = 302.21, *p* < 0.001). For Black males, no difference existed in measured stature and M10. Measured stature was greater than calculated stature from M8, M9, M11, M15, and MSH. For white males, measured stature did not differ from M15. Measured stature was greater than M8, M9, and M11, and less than M10 and MSH. When comparing races, no differences existed in measured stature or calculated stature using M8. Calculated stature was lower in Black vs white males for M9, M10, M11, M15, and MSH.

**Table 3:** Measured and MASH calculated statures (mean ± 1 standard deviation) for Black and white males and females.

		Stature (cm)	95% CI (cm)	vs measured		B vs W	
				*p*	ES (*d*)	*p*	ES (*d*)
Male							
Measured	B	175.6 ± 6.2	[174.9, 176.3]	–	–	0.090	0.09
	W	176.3 ± 6.1	[176.0, 176.6]	–	–		
M8	B	175.1 ± 5.9^#^	[174.4, 175.6]	< 0.001	0.07	0.357	0.05
	W	175.4 ± 6.1^#^	[175.1, 175.7]	< 0.001	0.11		
M9	B	173.0 ± 5.9^#^*	[172.3, 173.6]	< 0.001	0.36	< 0.001	0.20
	W	174.5 ± 6.2^#^	[174.2, 174.8]	< 0.001	0.24		
M10	B	175.8 ± 5.7*	[175.1, 176.4]	1.000	0.02	< 0.001	0.19
	W	177.2 ± 6.0^#^	[176.9, 177.5]	< 0.001	0.12		
M11	B	171.6 ± 6.2^#^*	[170.9, 172.3]	< 0.001	0.54	< 0.001	0.25
	W	173.4 ± 6.3^#^	[173.1, 173.8]	< 0.001	0.38		
M15	B	172.9 ± 5.7^#^*	[172.3, 173.6]	< 0.001	0.36	< 0.001	0.45
	W	176.3 ± 6.1	[176.0, 176.6]	1.000	0.01		
MSH	B	171.7 ± 5.8^#^*	[171.1, 172.4]	< 0.001	0.52	< 0.001	0.84
	W	178.0 ± 5.9^#^	[177.7, 178.3]	< 0.001	0.23		

		Stature (cm)	95% CI (cm)	vs measured		B vs W	
				*p*	ES (*r*)	*p*	ES (*r*)
Female							
Measured	B	163.1 ± 6.4	[162.6, 163.7]	–	–	0.059	0.06
	W	164.0 ± 5.6	[163.5, 164.4]	–	–		
F8	B	162.7 ± 6.1*	[162.2, 163.2]	0.784	0.01	0.010	0.08
	W	163.7 ± 5.3	[163.3, 164.1]	1.000	0.02		
F9	B	161.3 ± 6.0^#^*	[160.8, 161.9]	< 0.001	0.37	< 0.001	0.15
	W	163.3 ± 5.4^#^	[162.9, 163.7]	< 0.001	0.25		
F10	B	163.6 ± 6.3^#^	[163.1, 164.2]	0.008	0.11	0.443	0.02
	W	164.0 ± 5.6	[163.6, 164.4]	1.000	0.01		
F13	B	159.9 ± 6.0^#^*	[159.4, 160.5]	< 0.001	0.57	< 0.001	0.18
	W	162.2 ± 5.3^#^	[161.7, 162.6]	< 0.001	0.52		
F12	B	161.9 ± 6.1^#^*	[161.4, 162.5]	< 0.001	0.21	< 0.001	0.23
	W	164.9 ± 5.6^#^	[164.5, 165.4]	< 0.001	0.35		
FSH	B	157.8 ± 5.9^#^*	[157.2, 158.3]	< 0.001	0.74	< 0.001	0.42
	W	163.3 ± 5.3^#^	[162.8, 163.7]	< 0.001	0.15		

95% confidence intervals for statures are presented along with statistical differences and effect sizes of MASH calculated compared to measured stature within races (vs Measured) and between races for each stature measurement method (B vs W).

*MASH* maximum allowable standing height, *CI* confidence interval of the mean, *ES* effect size.

^#^Significant difference (*p* ≤ 0.05) between measured and calculated stature within each group. *A significant difference (*p* ≤ 0.05) in stature between sex-specific Black (B) and white (W) groups for a predictive equation.

For Black females, the main effect of stature significantly differed by equation (χ^2^_(6)_ = 1366.808, *p* < 0*.*001). No differences existed between measured stature and F8. Measured stature was greater than F9, F13, F12, and FSH but less than F10. For white females, the main effect of stature significantly differed by equation (χ^2^_(6)_ = 968.238, *p* < 0*.*001). No differences existed between measured stature and F8 or F10. Measured stature was greater than F9, F13, and FSH but less than F12. For race comparisons, Black and white males did not differ in measured stature or F10. Calculated statures for Black males were shorter than white males for F8, F9, F13, F12, and FSH (*p* ≤ 0.01).

**Figure 1:**
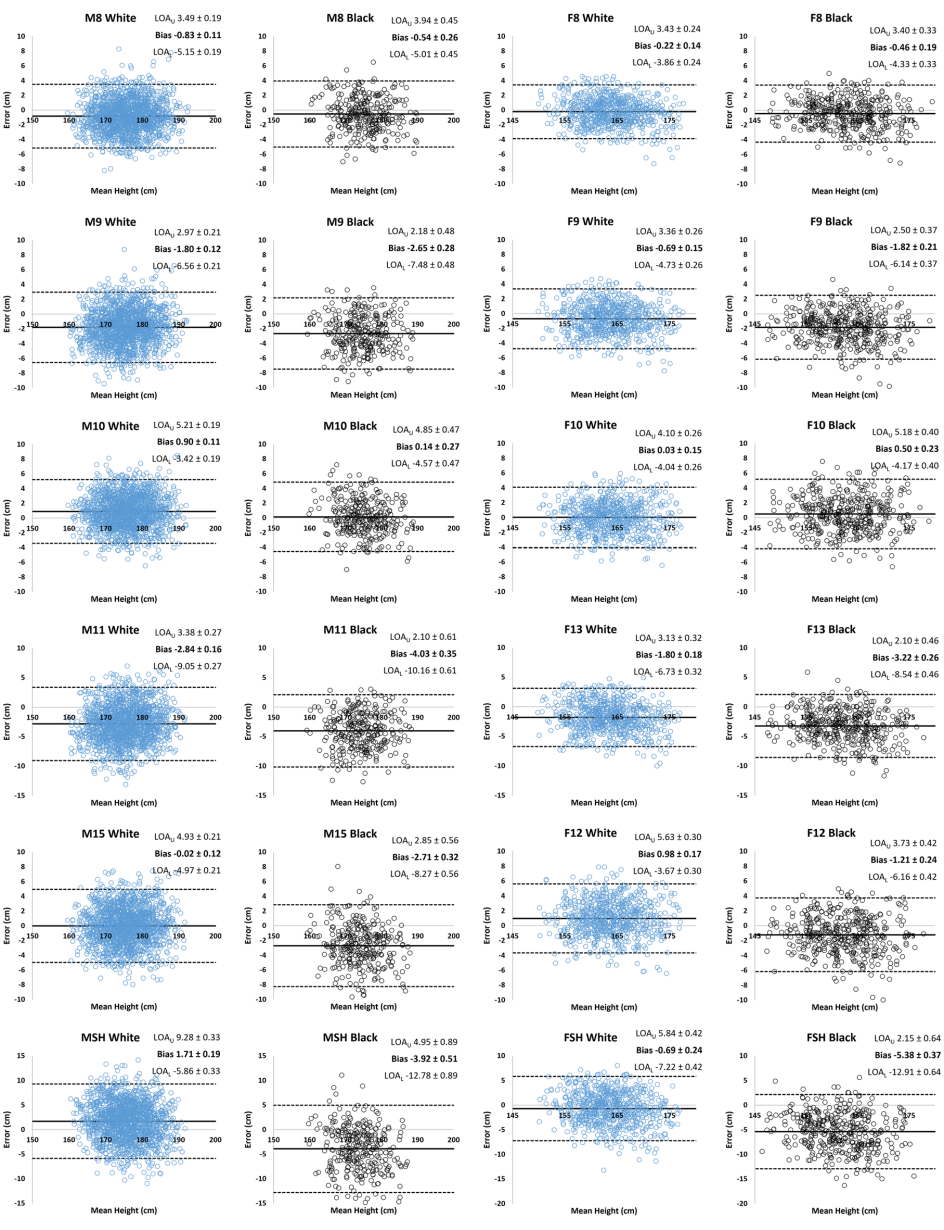
Bland–Altman plots of the mean of measured and estimated stature vs the difference (error), in cm, between the methods for each MASH equation for white (blue circles) and Black (black circles) males (columns 1 and 2) and females (columns 3 and 4). Mean bias errors (solid line) with upper and lower limits of agreement (LOA_U_, LOA_L_; dashed lines) are presented where values are mean ± 95% confidence interval.

R^2^, RMSE, mean bias error, bias error tolerance intervals, and coefficients of variation (CV) for each MASH equation are presented in Table 4. An expanded range of tolerance intervals can be found in the Supplementary Data. Linear regression indicated bias error and stature were negatively correlated for all equations and correlation coefficients generally increased in magnitude as MASH equations decreased in accuracy (Supplementary Data). For Bland–Altman analyses, all error comparisons had normal distributions (*p* > 0.05), except for white male equation M11 vs Stature (*p* = 0.03). In this case, the absolute differences of the errors and the means had a small negative correlation (r = − 0.08), and log transformations did not result in a normal distribution (*p* = 0.04), thus log transforms were not performed prior to calculating limits of agreement^48–50^. Bland–Altman plots (Fig. 1) show the mean bias and limits of agreement (LOA), i.e., the range within which 95% of all differences between the MASH-estimated and measured stature are likely to lie^48,51^. Plots indicated that LOA ranges increased as MASH equations decreased in accuracy.

**Table 4:** Correlation coefficients (R^2^) and error (RMSE and Bias) values of the MASH equations compared to measured stature for Black and white males and females.

		R^2^	RMSE (cm)	Bias (cm)	*p*	ES (*d*)	95% CI (cm)	95% TI limits (cm)	CV%
Male									
M8	B	0.86	2.27	− 0.54 ± 2.28*	0.04	0.04	[− 0.79, − 0.29]	(− 5.3, 4.3)	0.95
	W	0.87	2.32	− 0.83 ± 2.20			[− 0.94, − 0.72]	(− 5.3, 3.6)	0.95
M9	B	0.84	3.51	− 2.65 ± 2.47*	< 0.001	0.11	[− 2.93, − 2.38]	(− 7.8, 2.5)	1.47
	W	0.85	2.99	− 1.80 ± 2.43			[− 1.92, − 1.68]	(− 6.7, 3.1)	1.22
M10	B	0.85	2.33	0.14 ± 2.40*	< 0.001	0.10	[− 0.11, 0.39]	(− 4.9, 5.2)	0.98
	W	0.87	2.35	0.90 ± 2.20			[0.79, 1.00]	(− 3.6, 5.3)	0.96
M11	B	0.76	4.95	− 4.03 ± 3.13*	< 0.001	0.16	[− 4.39, − 3.67]	(− 10.6, 2.6)	2.08
	W	0.76	4.20	− 2.83 ± 3.17			[− 2.99, − 2.68]	(− 9.2, 3.6)	1.72
M15	B	0.79	3.80	− 2.71 ± 2.84*	< 0.001	0.35	[− 3.00, − 2.42]	(− 8.7, 3.3)	1.59
	W	0.83	2.49	− 0.02 ± 2.53			[− 0.15, 0.11]	(− 5.1, 5.1)	1.02
MSH	B	0.51	5.80	− 3.92 ± 4.52*	< 0.001	0.74	[− 4.36, − 3.47]	(− 13.4, 5.6)	2.43
	W	0.63	4.17	1.71 ± 3.86			[1.52, 1.91]	(− 6.1, 9.5)	1.70

		R^2^	RMSE (cm)	Bias (cm)	*P*	ES (*r*)	95% CI (cm)	95% TI limits (cm)	CV%
Female									
F8	B	0.91	2.02	− 0.46 ± 1.97*	0.05	0.06	[− 0.65, − 0.28]	(− 4.6, 3.6)	0.87
	W	0.89	1.85	− 0.22 ± 1.86			[− 0.36, − 0.08]	(− 4.0, 3.6)	0.81
F9	B	0.88	2.85	− 1.82 ± 2.21*	< 0.001	0.24	[− 2.02, − 1.62]	(− 6.4, 2.8)	1.24
	W	0.87	2.15	− 0.69 ± 2.06			[− 0.84, − 0.53]	(− 4.9, 3.5)	0.94
F10	B	0.87	2.43	0.50 ± 2.39*	0.002	0.09	[0.29, 0.71]	(− 4.5, 5.5)	1.06
	W	0.87	2.05	0.03 ± 2.08			[− 0.13, 0.19]	(− 4.2, 4.3)	0.89
F13	B	0.82	4.20	− 3.22 ± 2.71*	< 0.001	0.25	[− 3.47, − 2.97]	(− 8.9, 2.4)	1.83
	W	0.80	3.06	− 1.80 ± 2.52			[− 1.99, − 1.61]	(− 7.0, 3.4)	1.33
F12	B	0.85	2.79	− 1.21 ± 2.52*	< 0.001	0.39	[− 1.45. − 0.98]	(− 6.5, 4.0)	1.21
	W	0.83	2.54	0.98 ± 2.37			[0.80, 1.16]	(− 3.9, 5.8)	1.11
FSH	B	0.65	6.59	− 5.38 ± 3.84*	< 0.001	0.54	[− 5.72, − 5.04]	(− 13.4, 2.6)	2.88
	W	0.66	3.36	− 0.69 ± 3.33			[− 0.95, − 0.43]	(− 7.5, 6.1)	1.47

95% confidence intervals and 95% tolerance intervals for bias errors with coefficients of variation are also presented.

*MASH* maximum allowable standing height, *RMSE* root mean squared error, *ES* effect size, *CI* confidence interval of the mean, *TI* tolerance interval, *CV%* within-subjects coefficient of variation.

*A significant difference in Bias between sex-specific Black (B) and white (W) groups for a predictive equation. Bias values are mean ± 1 standard deviation.

For males, a significant main effect (*F*_(1,1897)_ = 155.44, *p* < 0*.*001) indicated bias error was significantly greater in Black compared to white males. A significant interaction existed between race and bias error (*F*_(2.15,4075.37)_ = 342.98, *p* < 0*.*001). Equations M8, M9, M11, and M15 had negative bias errors indicating they underestimated stature for both Black and white males. M8 underestimated stature significantly more for white than Black males, whereas stature was underestimated significantly more for Black than white males for equations M9, M11, and M15. M10 overestimated stature for both Black and white males, as indicated by positive bias errors, but to a greater extent in the white group. MSH significantly differed between the races and underestimated stature for Black males while overestimating stature for white males.

For females, F8, F9, F13, and FSH underestimated stature for both Black and white groups. Bias errors indicated stature was significantly more underestimated for Black than white females for F8, F9, F13, and FSH. F10 overestimated stature for both groups but more so for Black females. F12 underestimated stature for Black females but overestimated stature for white females.

## Discussion

A comparison of our data with Canda^12^ and Connick et al.^38^ highlights that different races and geographical groups can affect the accuracy and application of the MASH equations. Our Black and white populations were shorter in stature than Canda’s white Spanish population^12^ for both males and females and body segment proportionality differences were observed. The differences in our population’s anthropometric values produced poorer stature estimation performance demonstrated by smaller R^2^ values for all equations. Our Black populations generated greater RMSE values than Canda in 9 out of 10 equations (all but M10); however, two of the equations for each sex (M10, M15, F8, and F10) in our white populations produced lower RMSE values. This reflects that the MASH equations produce worse correlations while a majority of the equations produced greater stature prediction errors for Black and white American populations compared to a white Spanish population.

Four of the male MASH equations (M8, M9, M10, and M15) and three of the female equations (F8, F9, and F12) were validated by Connick et al.^38^ using white Australian and Asian Japanese populations. Our Black and white American males and females were shorter in stature compared to the white Australian groups but taller than the Asian Japanese groups. Varying body segment length to stature proportions were also observed. The equations generated lower R^2^ values in our Black males and females and white females than those reported by Connick et al.^38^ However, our white male population generated higher R^2^ values for 2 of the 4 equations (M10 and M15). Our Black and white females had RMSE values lower than Connick et al. for all three MASH equations. Our Black male group generated smaller RMSE values for 2 of the 4 equations (M8 and M10) while our white male group produced smaller RMSE values for 3 of the 4 equations (M8, M10, and M15). Connick et al. also reported bias errors that overestimated stature from all equations in both males and females^38^, which contrasts with our study’s underestimations of stature. Our data showed negative correlations between bias error and stature for each MASH equation where Connick et al.^38^ observed positive correlations for M8, F8, M9, and F9. Thus, these equations tend to overestimate stature for taller individuals in Australia or Japan but underestimate stature for taller individuals in the United States. Our correlation coefficients were also similar in magnitude or greater than prior studies. These comparisons show that anthropometric variability between Black and white Americans, white Australians, and Asian Japanese populations generates inconsistent R^2^ and error outcomes.

The purpose of this study was to determine if the currently used MASH equations validly calculate stature in Black and white populations from the United States. Statistical significance was used to determine equation validity. Thus, if an equation’s predicted stature significantly differed from the measured stature, the equation was labeled invalid. We recognize that a single, rigid definition may oversimplify determining an equation’s validity. Consequently, when a predictive equation was identified as statistically invalid, the mean bias error, RMSE, and effect sizes were evaluated to provide a multi-faceted approach to determine if the MASH equations provide “reasonable” stature estimations for practical purposes. LOAs provided further insights into the agreement between the estimated and measured stature values. Tolerance intervals, which present the range of stature prediction errors that encompass a certain percent of the population, were used to examine trends associated with the data spread and skew across the different populations and to supplement the interpretation of MASH equations’ predictive ability.

The first hypothesis stated that MASH equations would accurately calculate stature in Black Americans with no statistical difference compared to their measured stature. For males, this hypothesis was accepted for equation M10, indicating that the MASH equations will provide a similar estimate to stature for male Black American athletes with bilateral above-knee amputations and at least 1 intact upper arm and forearm. The hypothesis was rejected for equations M8, M9, M11, M15, and MSH. For females, this hypothesis was accepted for equation F8, indicating that the MASH equations will provide a similar estimate to stature for female Black American athletes with at least one intact thigh, upper arm, and forearm. This hypothesis was rejected for equations F9, F10, F13, F12, and FSH. The rejected equations can be considered invalid for estimating measured stature for male and female Black athletes.

The equations that resulted in significantly different calculations from measured stature had mean bias errors ranging from − 0.54 to − 4.03 cm for Black males and 0.50 to −5.38 cm for Black females. 95% LOAs ranged from 9.0 to 17.7 cm for Black males and 7.7 to 15.1 cm for Black females. 95% tolerance interval ranges spanned from 9.6 to 19.0 cm for Black males and 8.2 to 16.0 cm for Black females. The greater range of bias errors with smaller LOA and tolerance interval ranges for Black females indicate that the equations do a poorer job at estimating stature but were more consistent in their predictive ability relative to Black males. All five of the rejected equations underestimated stature for Black males. Four out of 5 rejected equations underestimated stature and 1 of 5 overestimated stature for Black females. An underestimation of stature greater than 5 cm likely has a greater impact on performance than an over- or underestimation of one-half cm. Our study included a large population that provided high statistical power. This resulted in bias errors that are sometimes small in magnitude yet statistically significant. It is not clear what amount of over- or underestimation of stature is acceptable since no agreed upon error limits currently exist for MASH equations; however, effect sizes and tolerance intervals provide additional perspective. The effect sizes for M8 were very small (*d* = 0*.*07) suggesting that the observed difference from measured stature may not be meaningful; however, the remaining significantly different equations (M9, M11, M15, and MSH) had effect sizes between *d* = 0*.*35 and 0.54, suggesting these equations on average meaningfully underestimate stature in Black American males. The confidence and tolerance intervals also indicated that bias errors were generally skewed toward underestimating stature, but overestimation also occurs. For females, small effect sizes were observed for F10 and F12 (*r* = 0*.*11 and 0.21, respectively), so these over- and underestimations of stature can be considered minimal. However, effect sizes for F9, F13, and FSH ranged from *r* = 0*.*37 to 0.74, suggesting they meaningfully underestimate stature for Black American females. Confidence and tolerance intervals confirmed that the equations tend to underestimate stature in this group, though upper tolerance limits indicate stature is occasionally overestimated.

Rejecting the use of a predictive equation that produces a small bias error may not be pragmatic. A bias error threshold of < 1 cm can be considered relatively small compared to mean measured statures over 175 cm and 163 cm for males and females, respectively. An RMSE threshold of < 3 cm represents the largest RMSE value reported for the MASH equations developed by Canda (2.97 cm)^12^. Using these exemplar thresholds of bias error < 1 cm and RMSE < 3 cm along with a small effect size (*d* ≤ 0.2; *r* < 0.3) on the rejected equations, we identified that equations M8 and F10 may be reasonable for use with Black males and Black females, respectively. The remaining statistically rejected equations (M9, M11, M15, and MSH for Black males and F9, F13, F12, and FSH for Black females) did not satisfy all three threshold requirements, indicating poor predictive performance. To improve the accuracy of these rejected equations, it is recommended that their corresponding bias errors be used to offset the MASH equation outcomes. Using an exemplar error tolerance threshold of ± 3 cm, 60% tolerance interval limits meet this requirement for both equations M8 and F10, indicating 40% of the population may fall outside of that error tolerance. However, bias error estimations were skewed toward underestimating stature, resulting in a lower percentage of the population meeting a particular error tolerance. Using a total error tolerance range of 6 cm shifts the tolerance interval limits to accounting for near 80% of the Black male and female populations. CVs for Black Americans ranged from 0.87 to 2.88%, suggesting the MASH equations performed reliably; however, low CV values alone do not indicate adequate reliability, and LOAs have been shown as the preferred method to assess intra-test variation^52,53^. Furthermore, CV methods should only be depended on if heteroscedasticity is present^50^, which was not the case for our data. The best performing MASH equation generated Bland–Altman LOAs where 95% of the errors fell within ± 3.9 cm. Thus, applying the ± 3 cm threshold results in all MASH equations being considered ambiguous in their ability to accurately estimate stature. This suggests that optimizing equations to reduce the predictive stature range will be beneficial, pending better understanding of the relationship between prosthesis lengthening and performance. Raising the threshold so the acceptable range of LOAs will fall within 5% of measured stature, LOA ranges greater than 8.2 cm for females and 8.8 cm for males can be considered ambiguous in their ability to accurately estimate stature. This leads to equation F8 being equivalent to measured stature for Black females but all other equations for Black Americans being ambiguous in their ability to accurately estimate stature.

The second hypothesis was that MASH equations would accurately calculate stature in white Americans with no difference compared to their measured stature. For males, this hypothesis was accepted for equation M15, indicating that the MASH equations provide a similar estimate to stature for male white American athletes with bilateral above-knee and below-elbow amputations with at least one intact upper arm. The hypothesis was rejected for equations M8, M9, M10, M11, and MSH, which can be considered invalid. For females, this hypothesis was accepted for equations F8 and F10, supporting the use of MASH equations in female white American athletes with at least one intact thigh, upper arm, and forearm as well as those with bilateral above-knee amputations and at least one intact upper arm and forearm. The hypothesis was rejected for equations F9, F13, F12, and FSH, so these equations may also be considered invalid for estimating stature.

The mean bias errors of the rejected equations ranged from 1.71 to − 2.83 cm for white males and 0.98 to − 1.80 cm for white females. CVs for white Americans ranged from 0.81 to 1.72% while 95% LOAs ranged from 8.6 to 15.1 cm for white males and 7.3 to 13.1 cm for white females. 95% tolerance interval ranges spanned from 8.9 to 15.6 cm for white males and 7.6 to 13.7 cm for white females. The MASH equations do a poorer job of estimating stature for white males than females as demonstrated by the greater range of average bias errors, LOAs, and tolerance interval ranges for males. Three of 5 rejected equations underestimated stature for males while 3 of 4 underestimated stature for females. Three equations, 2 male and 1 female, overestimated stature. For males, only equation M11 produced a small-medium effect size of *d* = 0*.*38 while the remaining equations had small effect sizes of *d* < 0.25. For females, small effect sizes were observed for F9 and FSH (*r* = 0*.*25 and 0.15, respectively) while medium and large effect sizes were observed for F12 (*r* = 0*.*35) and F13 (*r* = 0*.*52). As with the Black populations, it is not clear what amount of over- or underestimation of stature is acceptable given that the greatest mean overestimation error is less than 2 cm for white Americans and less than 3 cm for underestimation.

The multi-faceted method of using bias error (< 1 cm), RMSE (< 3 cm), and effect size (*d* ≤ 0.2; *r* < 0.3) thresholds along with considering tolerance intervals on the rejected equations as described earlier supports the reasonable use of M8 and M10 for white males and F9 for white females. The remaining statistically rejected equations (M9, M11, and MSH for white males and F13, F12, and FSH for white females) did not satisfy all three threshold requirements, and the LOAs and tolerance interval limits indicated greater bias error ranges. Likewise to the Black population outcomes, these rejected equations can be adjusted using the observed bias errors as offsets. The 60% tolerance interval limits met the ± 3 cm error tolerance threshold for equations M8 and M10, and the 70% limits met the threshold for equation F9. Using the total error range of 6 cm, each equation falls in the 80% tolerance interval limits, indicating less than 20% of the population fall outside of this requirement. LOAs indicate that 95% of errors between measured and estimated stature fell within at best ± 3.7 cm, suggesting that improved predictive equations may be needed to narrow this range. Raising the acceptable threshold range of LOAs to 5% measured stature (8.2 cm females, 8.8 cm males) leads to equations M8, M10, F8, F9, and F10 being equivalent to measured stature for white males and females.

Our third hypothesis proposed that the error between calculated and measured stature for Black Americans would be similar to white Americans. Four out of the 6 equations had larger bias errors and RMSE values for Black males and all six equations had larger bias errors and RMSE values for Black females compared to their white counterparts. Equations M8 and M10 had less bias and lower RMSE values for Black compared to white males. This hypothesis was rejected for both males and females as the errors of each predictive equation significantly differed between races. This indicates that the accuracy of the equations was race-dependent and anthropometric differences between the races influenced the predictive ability of the MASH equations.

Differences in bias error between Black and white males generally had small (M8, M9, M10, M11 *d* ≤ 0.16) to small-medium (M15 *d* = 0*.*35) effect sizes; however, the racial differences in bias error had a medium-large effect for MSH (*d* = 0*.*74). The MASH equations produced similar correlations between Black and white Americans with the exception of M15 and MSH, which had noticeably lower R^2^ values in Black males. These two equations were also the only ones where RMSE values between Black and white males differed by more than 1 cm. The tolerance interval ranges were consistently larger for Black males, but M10, M15 and MSH were the only equations where the difference in range exceeded 1 cm. Taken together, these outcomes suggest that equations M15 and MSH did not predict stature similarly for Black and white American males. One can argue that the remaining equations (M8, M9, M10, M11) perform similarly between the races due to smaller effect sizes and RMSE value differences; however, the systematic bias where the equations consistently generate larger errors for Black males remains a concern.

Effect sizes for the differences in bias error between Black and white females were very small for F8 and F10 (*r* ≤ 0.09) and small for F9 and F13 (*r* = 0*.*24–0.25) but medium for F12 (*r* = 0*.*39) and large for FSH (*r* = 0*.*54). While R^2^ of the MASH equations was similar between female groups for each equation, all RMSE values were greater for Black females, where F13 differed by more than 1 cm and FSH differed by greater than 3 cm. Tolerance interval ranges were greater for Black females for every equation, where F10, F13, and FSH had range differences greater than or equal to 1 cm. The tolerance limits were shifted to more negative for F9, F13, F12, and FSH. These data suggest that equations F13, F12, and FSH do not predict stature with the same accuracy for both Black and white females. The female equations F8, F9, and F10 had smaller effect sizes and RMSE value differences, thus it can be debated that these equations predict stature similarly between the races. Similar to the male data, systematic bias was evident where the female equations generated greater errors for Black females.

Overall, two MASH equations for Black Americans and three for white Americans were statistically valid and two additional equations for Black Americans and three for white Americans were considered reasonable for use. The remaining eight equations for Black Americans and six equations for white Americans were invalid (Table 5). Furthermore, the observed ranges of bias error tolerance limits highlights that MASH equations have a fairly wide predictive ability for all groups. The best performing equations (i.e., those determined as valid or reasonable for use) resulted in 95% of individuals falling within bias error tolerance limit ranges between 7.6 and 10.2 cm (Table 4). As demonstrated by the lower and upper tolerance limits of the best performing equation (F8 for white females), one athlete could have stature underestimated by 4 cm and another athlete could have stature overestimated by 3.6 cm, resulting in an effective height difference of 7.6 cm. Using a notional threshold of 6 cm as an acceptable predictive error range, the best performing MASH equation for each sex-specific Black and white group accounts for ~ 75% (M10 for Black and M15 for white males) to ~ 85% (F8 for Black and white females) of the group populations. To reduce the potential for stature estimation differences between athletes, MASH equations should ideally generate more consistent stature predictions with lower tolerance interval ranges.

**Table 5:** Summary of MASH equations and their validity.

	Valid	Invalid, but reasonable for use	Invalid, recommend new equation development
Black male	M10	M8	M9, M11, M15, MSH
White male	M15	M8, M10	M9, M11, MSH
Black female	F8	F10	F9, F13, F12, FSH
White female	F8, F10	F9	F13, F12, FSH

*MASH* maximum allowable standing height.

The findings from our study justify the need to develop new MASH equations for Black and white Americans. Although the development of new equations was beyond the scope of this study, our data provide the groundwork and direction for future studies. Thresholds for acceptable error levels for the MASH equations are needed as guidance for future equation development and validation efforts. Such thresholds should be informed by research investigating performance advantages caused by prosthesis lengthening. Ideally the MASH equations will generalize for all racial and geographical populations. The equations should be validated with additional racial and geographical groups to determine the need to either develop race- and geography-specific equations for the populations whose statures were not validly calculated by the current MASH equations or develop new generalizable MASH equations that will apply to all para-athletes regardless of race or geography.

Several limitations should be considered when interpreting data from this study. Our study used anthropometric measurement procedures that followed US Army and Marine Corps guidelines^46,47^, whereas the MASH equations were developed using International Society for the Advancement of Kinanthropometry (ISAK) measurement procedures^54^. Differences between the procedures include the forearm measurement being performed with the palm facing forward instead of the palm facing the thigh, which could cause a minor difference in forearm length. The thigh length measurement was calculated by subtracting tibiale mediale height from the trochanterion height instead of directly measuring the distance between the trochanterion and tibiale laterale, which can result in a slightly shorter thigh length. Differences in forearm and thigh lengths could influence the predictive ability of the MASH equations and their comparison to measured stature; however, these data will not impact the comparisons of Black vs white populations presented here. It is noted that during MASH Measurement Sessions, where body segment lengths are measured for para-athletic competitions, the procedures for athletes with amputations may deviate from ISAK procedures due to setup and anatomical landmarking limitations.

This study is limited to investigating Black and white populations from the United States, thus it is recommended to validate MASH equations with additional racial and geographical populations.

The stature and limb proportions of military personnel used in this study may not represent elite athletes. On average, our populations were shorter than Canda and Connick et al.’s white athletes, but taller than Connick et al.’s Asian Japanese athletes^12,38^. The different statures of each group could impact geographical comparisons, but also highlights potential regional differences. Validating anthropometric data using elite athletes will potentially improve the accuracy of MASH equations, and future studies should directly compare MASH predictions using athletes from different geographic locales.

Finally, in practice, pure error values between 1.73 and 2.97 cm are added to each MASH stature estimation based on Canda’s^12^ observed bias errors. Adding these values to our stature estimates yields overestimated mean statures in most cases, but does not change comparative analyses. Pure error values were not included in our analyses so we could generate unbiased stature estimations from the MASH equations and directly compare to the literature, which also does not use pure error values as inputs. The mean bias errors observed in this study (Table 4, Fig. 1) can be offset from each MASH equation result to provide more accurate stature estimations for Black and white para-athletes from the United States.

## Conclusion

While the existing MASH equations are the best available for stature estimation, this study suggests that while some MASH equations were valid or were considered reasonable for use (Table 5), a majority of the equations did not accurately estimate stature in Black and white males and females from the United States. Furthermore, bias errors significantly differed between Black and white males and females for every equation, and a systematic bias was evident where the MASH equations consistently generated greater errors for Black compared to white Americans. This study confirms that race and geography impact the validity of the MASH equations.

Several recommendations related to stature estimation can be made to improve the accuracy, validity, and generalizability of MASH equations and consequently enhance fairness in competitions for para-athletes: (1) use the bias errors from this study as offsets to MASH equation outcomes for Black and white para-athletes; (2) validate the MASH equations with additional racial and geographical populations; (3) identify acceptable MASH error thresholds for average bias and LOA ranges based on the prosthesis lengthening that provides a significant performance advantage; and (4) develop new MASH equations with a large, racially and geographically diverse group of athletes to minimize population biases.

## Methods

Study demographic and anthropometric data were obtained from the ANSUR II database^45–47^, a publicly available comprehensive measurement study performed by anthropometric experts on US Army service members. Since MASH equations are most commonly applied to adult elite athletes, military service members who are often considered “tactical athletes” can well represent an athletic population. Furthermore, the database was screened to include male and female participants between 18 and 35 years old with a body mass index (BMI) less than 30 kg/m^2^. Metrics of interest included age, body mass, stature, sitting height, thigh length, upper arm length, forearm length, and arm span. Body segment length to stature ratios were then calculated for sitting height, thigh length, upper arm length, forearm length, and arm span.

### Anthropometric measurements

Stature and body segment measurements in the ANSUR II database were obtained following the US Army and Marine Corps guidelines described in Gordon et al.^47^ and Hotzman et al.^46^. Brief setup, anatomical landmark, and measuring descriptions are as follows:

#### Stature

The vertical distance from a standing surface to the top of the head was measured with an anthropometer. The participant stood erect with the head in the Frankfurt plane, heels together, and weight distributed equally on both feet.

#### Sitting height

The vertical distance between the sitting surface and the top of the head was measured with an anthropometer. The participant sat erect with the head in the Frankfurt plane, thighs parallel, knees flexed 90°, and feet in line with the thighs.

#### Thigh length

Thigh length was calculated as the trochanterion height minus tibiale mediale height. Trochanterion height was the vertical distance between the standing surface and right trochanterion (superior point of the greater trochanter) landmark while tibiale mediale height was the vertical distance between the standing surface and right tibiale mediale (superior point on the medial condyle of the tibia) landmark. The participant stood erect with heels together and weight distributed equally on both feet, and the vertical distances were measured with an anthropometer.

#### Upper arm length

The distance between the right acromion (lateral border of the acromial process) and radiale (superior point on the outside edge of the radius) landmarks was measured with a beam caliper held parallel to the long axis of the arm. The participant stood erect with shoulders and upper extremities relaxed and the palms facing the thighs.

#### Forearm length

The distance between the right radiale and stylion (distal point of the radius) landmarks was measured with a beam caliper held parallel to the long axis of the forearm. The participant stood with the arms relaxed at the sides, and the hand and fingers were held straight in line with the long axis of the forearm with the palm facing forward.

#### Arm span

The distance between the tips of the middle fingers of both hands was measured on a wall chart. The participant stood erect with their back against a wall-mounted scale, heels together, and both arms and hands were stretched horizontally along the wall.

### MASH equations

MASH is calculated for each para-athlete by using 1 of 6 sex-specific equations that predict stature using combinations of limb and body segment lengths available on the athlete^11^. Equations (1–12) detail the current MASH equations:

1 $${\text{M}}8 = -5.272 + (0.998*\text{sitting height}) + (0.855*{\text{thigh}}) + (0.882*\text{upper arm}) + (0.820*{\text{forearm}})$$

2 $${\text{M}}9 = -6.059 + (1.059*\text{sitting height}) + (0.953*{\text{thigh}}) + (1.233*\text{upper arm})$$

3 $${\text{M}}10 = -5.857 + (1.116*\text{sitting height}) + (1.435*\text{upper arm}) + (1.189*{\text{forearm}})$$

4 $${\text{M}}11 = -7.517 + (1.283*\text{sitting height}) + (1.439*{\text{thigh}})$$

5 $${\text{M}}15 = -7.217 + (1.231*\text{sitting height}) + (2.075*\text{upper arm})$$

6 $$\text{MSH }=\text{ Sitting height }/ 0.52$$

7 $${\text{F}}8 = -0.126 + (1.022*\text{sitting height}) + (0.698*{\text{thigh}}) + (0.899*\text{upper arm}) + (0.779*{\text{forearm}})$$

8 $${\text{F}}9 = -0.686 + (1.061*\text{sitting height}) + (0.814*{\text{thigh}}) + (1.237*\text{upper arm})$$

9 $${\text{F}}10 = -4.102 + (0.509*\text{arm span}) + (0.966*\text{sitting height})$$

10 $${\text{F}}13 = 0.685 + (1.246*\text{sitting height}) + (1.306*{\text{thigh}})$$

11 $${\text{F}}12 = -1.663 + (1.184*\text{sitting height}) + (2.039*\text{upper arm})$$

12 $$\text{FSH }=\text{ Sitting height }/ 0.533$$

Five of the 6 sex-specific MASH equations are described with a letter and number moniker, where M and F represent male and female specific equations, respectively, and the number represents the accuracy and precision ranking from Canda^12^ in order of greatest to least R^2^ value. The remaining MASH equations are labelled MSH and FSH as they are derived from Contini^13^ using sitting height.

Statures were calculated from each MASH equation. R^2^, RMSE, and bias error values were then determined for each sex-based equation within Black and white groups.

### Statistics

Assumptions for parametric statistical tests were assessed by examining the data for skew/kurtosis, outliers, normal distribution, homogeneity of variance, and sphericity. Outliers were identified using studentized residuals greater than ± 3 standard deviations and were removed from the dataset. Male data passed assumption testing with only minor, correctable violations. The homogeneity of variance assumption was violated for all variables in the female dataset (Levene’s test, *p* < 0*.*05 for all). Therefore, parametric statistics were used to assess the male data and non-parametric statistics were used for the female data comparisons. Tolerance intervals were calculated for bias error measurements to estimate the range of error values for a specified portion of the population. For predictive equations, tolerance intervals would typically be created using the limits of accepted error or performance. Because no agreed upon acceptable error limits exist for MASH equations, the tolerance intervals were constructed such that the lower and upper limits encompassed 95%, 90%, 80%, 70%, 60%, and 50% of the population with 95% confidence. Normal distributions were verified for both male and female stature and bias errors, so parametric calculations of tolerance intervals were used.

Bland–Altman plots were constructed to assess the agreement between measured and estimated stature, where the mean of the measurements was plotted against the difference of the methods (i.e., error)^48^. Errors were assessed for normality using the Anderson–Darling test^49^. If errors were not normally distributed, heteroscedasticity was assessed by correlating the absolute errors and individual means, where a positive correlation indicated the need to log transform data prior to calculating limits of agreement^48–50^. Within-subject coefficients of variation (CV%) were calculated for each MASH equation^55^. Within-subject stature variance, *s*^2^, was calculated for each subject as (MASH – Measured)^2^/2. Within-subject variance was divided by the subject’s squared mean stature from each method (*s*^2^/*m*^2^), and the mean was calculated across all subjects for each group. Within-subject CV% was calculated as the square root of the mean of *s*^2^/*m*^2^ multiplied by 100.

Study population descriptive data, body segment lengths, and length to stature ratios were compared between Black and white groups using Student’s independent t-tests for males and Mann–Whitney U tests for females with Bonferroni adjustments for multiple comparisons. For males, a 2-way (2 × 7) Mixed ANOVA determined the effect of Race on Stature. Race (Black, white) was treated as a between factors variable. Stature was treated as a within factors variable (Measured Stature, M8, M9, M10, M11, M15, and MSH). A 2-way (2 × 6) Mixed ANOVA was performed to compare the effect of Race on Bias Error. Race (Black, white) was treated as a between factors variable, and Bias Error was treated as a within factors variable (M8, M9, M10, M11, M15, and MSH). When the full factorial models identified significant differences, 1-way ANOVAs and pairwise comparisons with Bonferroni adjustments determined which conditions differed from each other. Greenhouse–Geisser estimates and Games Howell post hoc tests were used to address the minor violations of sphericity and homogeneity of variance. Effect sizes were calculated using Cohen’s *d* (mean difference divided by mean squared error) where *d* = 0*.*2, *d* = 0*.*5, and *d* = 0*.*8 are considered small, medium, and large effects, respectively^56^.

For females, Mann Whitney U tests compared the effect of Race (Black, white) on Stature (Measured Stature, F8, F9, F10, F13, F12, and FSH) and on Bias Error (F8, F9, F10, F13, F12, and FSH). Within each race, Friedmans tests determined whether differences existed between stature calculations (Measured Stature, F8, F9, F10, F13, F12, and FSH). All assumptions for use of Mann–Whitney U and Friedmans tests were met, and Bonferroni corrections for multiple comparisons were employed. Effect sizes, *r*, were calculated as the absolute value of the z-statistic divided by the square root of the number of samples, where *r* < 0.3, *r* = 0*.*3–0.5, and *r* > 0.5 were considered small, medium, and large effects, respectively^57^. Statistical analyses were performed using SPSS 29.0 (IBM Inc., Armonk, NY, USA), and significance levels were set at α = 0.05.

### Supplementary Information

Supplementary Information.

## Data Availability

The datasets analyzed during the current study are publicly available in the Defense Centers for Public Health—Aberdeen Anthropometric Database repository, https://phc.amedd.army.mil/topics/workplacehealth/ergo/Pages/Anthropometric-Database.aspx. The publicly available database overview file along with the male and female database files can be downloaded directly through the following links: https://phc.amedd.army.mil/PHC%20Resource%20Library/ANSURIIDatabasesOverview.pdf, https://phc.amedd.army.mil/PHC%20Resource%20Library/ANSURIIMALEPublic.csv, https://phc.amedd.army.mil/PHC%20Resource%20Library/ANSURIIFEMALEPublic.csv.
