# Supplementary material for: Race and geography impact validity of maximum allowable standing height equations for para-athletes
Source: Sci Rep. 2024 Mar 19;14:6551. doi: 10.1038/s41598-024-56597-y (PMC10951375; doi:10.1038/s41598-024-56597-y)
Supplement: Supplementary file 1 — Supplementary Information. [file 41598_2024_56597_MOESM1_ESM.docx]

**Supplementary Data**

**Supplementary Table 1.** Correlation coefficients (R^2^) and error (RMSE and Bias) values of the MASH equations compared to measured stature for Black and white males and females. 95% confidence intervals and a range of tolerance intervals for bias errors are also presented.

|  |  |  | **R^2^** | **RMSE**  **(cm)** | **Bias**  **(cm)** | ***p*** | **ES**  **(*d*)** | **95% CI**  **(cm)** | **95% TI Limits**  **(cm)** | **90% TI Limits**  **(cm)** | **80% TI Limits**  **(cm)** | **70% TI Limits**  **(cm)** | **60% TI Limits**  **(cm)** | **50% TI Limits**  **(cm)** |
| --- | --- | --- | --- | --- | --- | --- | --- | --- | --- | --- | --- | --- | --- | --- |
| **Male** | **M8** | B | 0.86 | 2.27 | -0.54 ± 2.28* | .04 | 0.04 | [-0.79, -0.29] | (-5.3, 4.3) | (-4.6, 3.5) | (-3.7, 2.6) | (-3.1, 2.0) | (-2.6, 1.5) | (-2.2, 1.1) |
|  |  | W | 0.87 | 2.32 | -0.83 ± 2.20 |  |  | [-0.94, -0.72] | (-5.3, 3.6) | (-4.6, 2.9) | (-3.7, 2.1) | (-3.2, 1.5) | (-2.7, 1.1) | (-2.4, 0.7) |
|  | **M9** | B | 0.84 | 3.51 | -2.65 ± 2.47* | <.001 | 0.11 | [-2.93, -2.38] | (-7.8, 2.5) | (-7.0, 1.7) | (-6.0, 0.7) | (-5.4, 0.1) | (-4.9, -0.4) | (-4.4, -0.9) |
|  |  | W | 0.85 | 2.99 | -1.80 ± 2.43 |  |  | [-1.92, -1.68] | (-6.7, 3.1) | (-5.9, 2.3) | (-5.0 ,1.4) | (-4.4, 0.8) | (-3.9, 0.3) | (-3.5, -0.1) |
|  | **M10** | B | 0.85 | 2.33 | 0.14 ± 2.40* | <.001 | 0.10 | [-0.11, 0.39] | (-4.9, 5.2) | (-4.1, 4.4) | (-3.2, 3.4) | (-2.5, 2.8) | (-2.0, 2.3) | (-1.6, 1.9) |
|  |  | W | 0.87 | 2.35 | 0.90 ± 2.20 |  |  | [0.79, 1.00] | (-3.6, 5.3) | (-2.8, 4.6) | (-2.0, 3.8) | (-1.5, 3.2) | (-1.0, 2.8) | (-0.6, 2.4) |
|  | **M11** | B | 0.76 | 4.95 | -4.03 ± 3.13* | <.001 | 0.16 | [-4.39, -3.67] | (-10.6, 2.6) | (-9.6, 1.5) | (-8.3, 0.3) | (-7.5, -0.5) | (-6.9, -1.2) | (-6.3, -1.8) |
|  |  | W | 0.76 | 4.20 | -2.83 ± 3.17 |  |  | [-2.99, -2.68] | (-9.2, 3.6) | (-8.2, 2.5) | (-7.0, 1.4) | (-6.2, 0.6) | (-5.6, -0.1) | (-5.0, -0.6) |
|  | **M15** | B | 0.79 | 3.80 | -2.71 ± 2.84* | <.001 | 0.35 | [-3.00, -2.42] | (-8.7, 3.3) | (-7.7, 2.3) | (-6.6, 1.2) | (-5.9, 0.4) | (-5.3, -0.1) | (-4.8, -0.7) |
|  |  | W | 0.83 | 2.49 | -0.02 ± 2.53 |  |  | [-0.15, 0.11] | (-5.1, 5.1) | (-4.3, 4.3) | (-3.4, 3.3) | (-2.7, 2.7) | (-2.2, 2.2) | (-1.8, 1.7) |
|  | **MSH** | B | 0.51 | 5.80 | -3.92 ± 4.52* | <.001 | 0.74 | [-4.36, -3.47] | (-13.4, 5.6) | (-11.9, 4.1) | (-10.1, 2.3) | (-9.0, 1.1) | (-8.0, 0.2) | (-7.2, -0.6) |
|  |  | W | 0.63 | 4.17 | 1.71 ± 3.86 |  |  | [1.52, 1.91] | (-6.1, 9.5) | (-4.8, 8.3) | (-3.4, 6.8) | (-2.4, 5.8) | (-1.6, 5.1) | (-1.0, 4.4) |
|  |  |  | **R^2^** | **RMSE**  **(cm)** | **Bias**  **(cm)** | ***p*** | **ES**  **(*r*)** | **95% CI**  **(cm)** | **95% TI Limits**  **(cm)** | **90% TI Limits (cm)** | **80% TI Limits**  **(cm)** | **70% TI Limits**  **(cm)** | **60% TI Limits**  **(cm)** | **50% TI Limits**  **(cm)** |
| **Female** | **F8** | B | 0.91 | 2.02 | -0.46 ± 1.97* | .05 | 0.06 | [-0.65, -0.28] | (-4.6, 3.6) | (-3.9, 3.0) | (-3.1, 2.2) | (-2.6, 1.7) | (-2.2, 1.3) | (-1.9, 1.0) |
|  |  | W | 0.89 | 1.85 | -0.22 ± 1.86 |  |  | [-0.36, -0.08] | (-4.0, 3.6) | (-3.4, 3.0) | (-2.7, 2.3) | (-2.2, 1.8) | (-1.9, 1.4) | (-1.5, 1.1) |
|  | **F9** | B | 0.88 | 2.85 | -1.82 ± 2.21* | <.001 | 0.24 | [-2.02, -1.62] | (-6.4, 2.8) | (-5.7, 2.0) | (-4.8, 1.2) | (-4.2, 0.6) | (-3.8, 0.2) | (-3.4, -0.2) |
|  |  | W | 0.87 | 2.15 | -0.69 ± 2.06 |  |  | [-0.84, -0.53] | (-4.9, 3.5) | (-4.2, 2.9) | (-3.5, 2.1) | (-2.9, 1.6) | (-2.5, 1.1) | (-2.1, 0.8) |
|  | **F10** | B | 0.87 | 2.43 | 0.50 ± 2.39* | .002 | 0.09 | [0.29, 0.71] | (-4.5, 5.5) | (-3.7, 4.7) | (-2.7, 3.8) | (-2.1, 3.1) | (-1.6, 2.6) | (-1.2, 2.2) |
|  |  | W | 0.87 | 2.05 | 0.03 ± 2.08 |  |  | [-0.13, 0.19] | (-4.2, 4.3) | (-3.5, 3.6) | (-2.8, 2.8) | (-2.2, 2.3) | (-1.8, 1.9) | (-1.4, 1.5) |
|  | **F13** | B | 0.82 | 4.20 | -3.22 ± 2.71* | <.001 | 0.25 | [-3.47, -2.97] | (-8.9, 2.4) | (-8.0, 1.5) | (-6.9, 0.5) | (-6.2, -0.2) | (-5.6, -0.8) | (-5.2, -1.3) |
|  |  | W | 0.80 | 3.06 | -1.80 ± 2.52 |  |  | [-1.99, -1.61] | (-7.0, 3.4) | (-6.1, 2.5) | (-5.2, 1.6) | (-4.5, 0.9) | (-4.0, 0.4) | (-3.6, 0.0) |
|  | **F12** | B | 0.85 | 2.79 | -1.21 ± 2.52* | <.001 | 0.39 | [-1.45. -0.98] | (-6.5, 4.0) | (-5.6, 3.2) | (-4.7, 2.2) | (-4.0, 1.6) | (-3.5, 1.0) | (-3.0, 0.6) |
|  |  | W | 0.83 | 2.54 | 0.98 ± 2.37 |  |  | [0.80, 1.16] | (-3.9, 5.8) | (-3.1, 5.1) | (-2.2, 4.2) | (-1.6, 3.6) | (-1.1, 3.1) | (-0.7, 2.7) |
|  | **FSH** | B | 0.65 | 6.59 | -5.38 ± 3.84* | <.001 | 0.54 | [-5.72, -5.04] | (-13.4, 2.6) | (-12.1, 1.3) | (-10.6, -0.2) | (-9.6, -1.2) | (-8.8, -1.9) | (-8.1, -2.6) |
|  |  | W | 0.66 | 3.36 | -0.69 ± 3.33 |  |  | [-0.95, -0.43] | (-7.5, 6.1) | (-6.4, 5.0) | (-5.2, 3.8) | (-4.3, 2.9) | (-3.6, 2.2) | (-3.0, 1.7) |
| * indicates a significant difference in Bias between sex-specific Black (B) and white (W) groups for a predictive equation. Bias values are mean ± 1 standard deviation. | | | | | | | | | | | | | | |
| Abbreviations: MASH = maximum allowable standing height; RMSE = root mean squared error; ES = effect size; CI = confidence interval of the mean; TI = tolerance interval. | | | | | | | | | | | | | | |


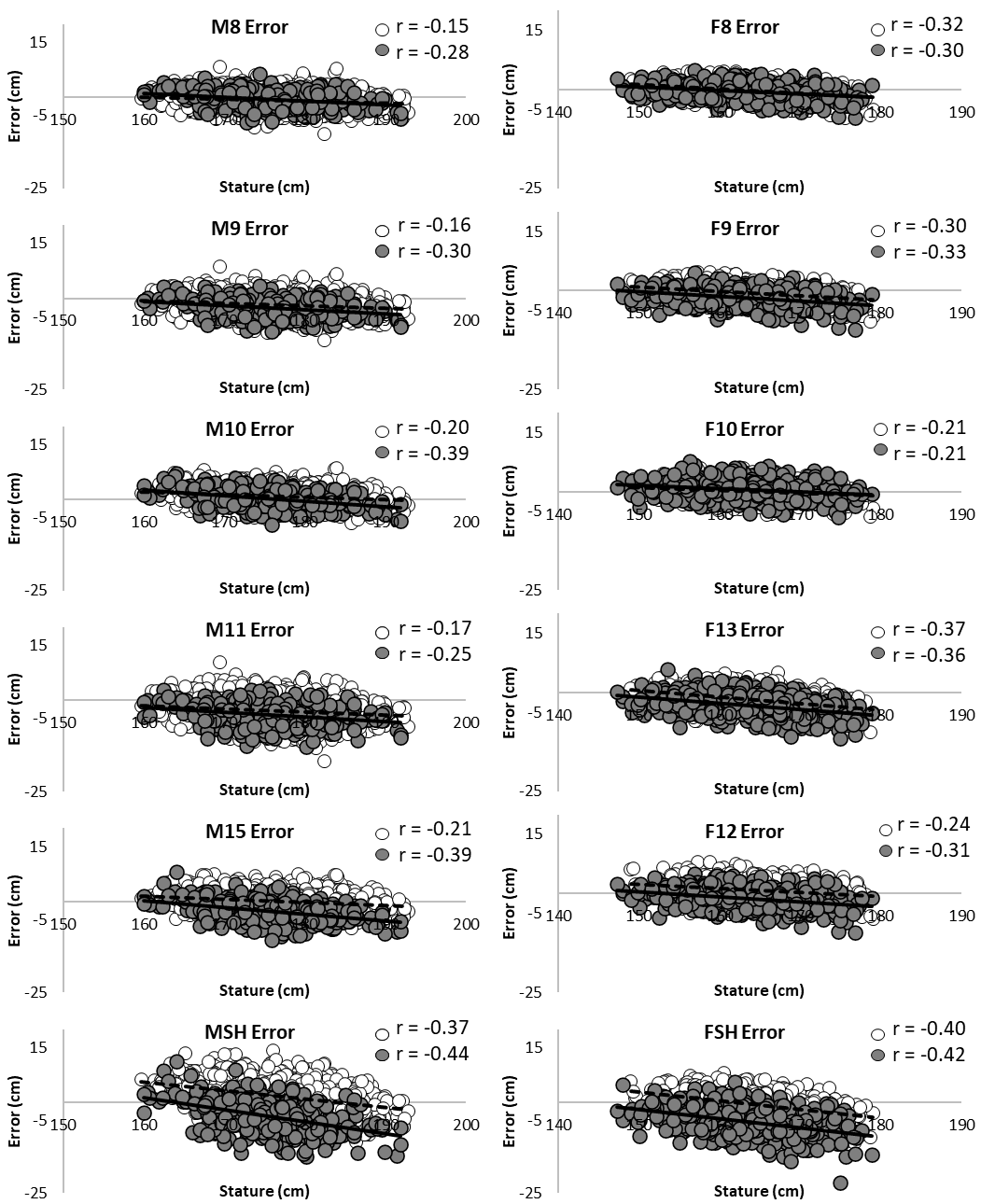


**Supplementary Figure 1**. Bias error vs measured stature for each of the MASH equations for males (left column) and females (right column). Closed and open circles represent Black and white individuals, respectively. The solid (Black) and dashed (white) lines represent the linear trendlines of each group.
